# Selective delivery of low-affinity IL-2 to PD-1^+^ T cells rejuvenates antitumor immunity with reduced toxicity

**DOI:** 10.1172/JCI153604

**Published:** 2022-02-01

**Authors:** Zhenhua Ren, Anli Zhang, Zhichen Sun, Yong Liang, Jianfeng Ye, Jian Qiao, Bo Li, Yang-Xin Fu

**Affiliations:** 1 Department of Pathology,; 2 Department of Pharmacology, Harold C. Simmons Comprehensive Cancer Center, and; 3 Lyda Hill Department of Bioinformatics, UT Southwestern Medical Center, Dallas, Texas, USA.; 4 Department of Basic Medical Science, Tsinghua University, Beijing, China.

**Keywords:** Immunology, Cancer immunotherapy

## Abstract

PD-1 signaling on T cells is the major pathway that limits T cell immunity, but the efficacy of anti–PD-1 therapy has been limited to a small proportion of patients with advanced cancers. We fortuitously observed that anti–PD-1 therapy depends on IL-2 signaling, which raises the possibility that a lack of IL-2 limits anti–PD-1–induced effector T cell expansion. To selectively deliver IL-2 to PD-1^+^CD8^+^ tumor-infiltrating lymphocytes (TILs), we engineered a low-affinity IL-2 paired with anti–PD-1 (PD-1–laIL-2), which reduced affinity to peripheral Treg cells but enhanced avidity to PD-1^+^CD8^+^ TILs. PD-1–laIL-2 exerted better tumor control and lower toxicity than single or mixed treatments. Mechanistically, PD-1–laIL-2 could effectively expand dysfunctional and tumor-specific CD8^+^ T cells. Furthermore, we discovered that presumably dysfunctional PD-1^+^TIM3^+^ TILs are the dominant tumor-specific T cells responding to PD-1–laIL-2. Collectively, these results highlight that PD-1–laIL-2 can target and reactivate tumor-specific TILs for tumor regression as a unique strategy with stronger efficacy and lower toxicity.

## Graphical abstract

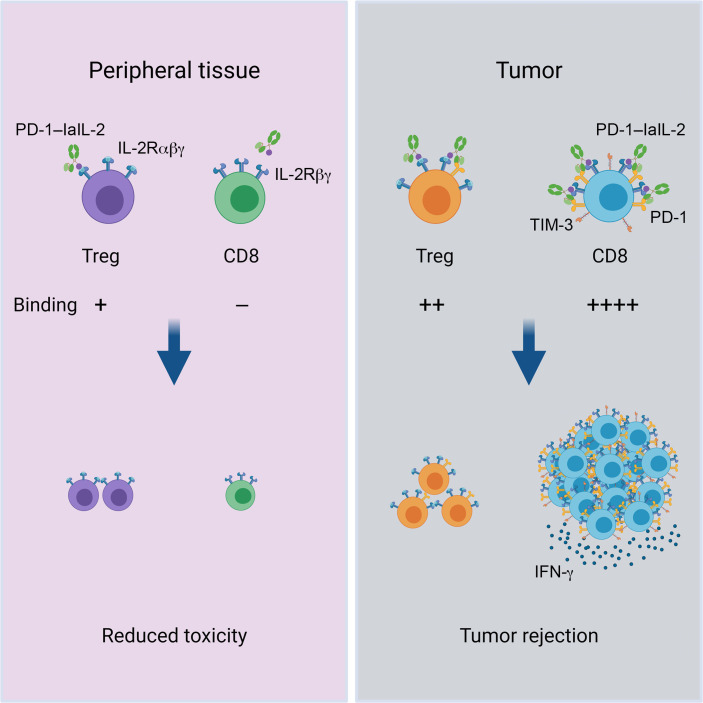

## Introduction

Increased levels of tumor-infiltrating lymphocytes (TILs) are associated with improved survival in patients with cancer (1–3). Anti–PD-1/PD-L1–based cancer immunotherapies have revolutionized the treatment of cancers (4–8), and TIL abundance can be used as a prediction marker for immunotherapy responsiveness (9, 10). Even though PD-1/PD-L1 blockade can release the brake on the T cell response, T cells are not fully functional and are limitedly expanded in the tumor (11, 12). Most patients either fail to respond or develop adaptive resistance after an initial response (13–15). Importantly, the role of T cell–associated cytokines in the tumor microenvironment for anti–PD-1/PD-L1 responsiveness has not been fully studied. It is possible that additional T cell–driven cytokine therapy might overcome PD-1 therapy resistance.

IL-2 is an important T cell growth factor for T cell proliferation (16). The IL-2 receptor has 3 subunits—IL-2Rα (CD25), IL-2Rβ (CD122), and IL-2Rγ—expressed on T cells and NK cells (17, 18). The IL-2Rα subunit is mainly expressed on Treg cells. Therefore, low doses of IL-2 treatment lead to the production of more Treg cells than CD8^+^ T cells (19, 20). High-dose IL-2 treatment can overcome Treg-associated IL-2 trapping and allow extra IL-2 to activate TILs for treating metastatic renal cell carcinoma and melanoma (21–24). However, patients who respond to high-dose IL-2 treatment frequently suffer from intolerable toxicities (25), which limits its clinical use. Extensive efforts have been made to generate mutants that either reduce the binding to IL-2Rα on Tregs (26) or increase the binding to IL-2Rβ on effector cells (27, 28).

How to target IL-2 to tumor-specific T cells remains a challenge in IL-2 cancer immunotherapy. Most studies focus on using antitumor antigens to bring IL-2 into tumor tissues (29–31). For example, fibronectin, which is expressed abundantly around neovascular structures in tumors, has been applied for treatment (32–34). Several other targets have been assessed for their ability to deliver IL-2 to the tumor site; these include carcinoembryonic antigen (CEA) and fibroblast activation protein (FAP) (35–38). However, these strategies can only bring IL-2 to tumor sites, and strategies to target IL-2 effectively and specifically to intratumoral effector T cells instead of other undesired cells have not been discovered thus far.

Here, we designed a fusion protein (IL-2 linked to an anti–PD-1 antibody) to target TILs, as TILs express more PD-1 than other cells. To reduce the binding of IL-2 to Tregs, we selected a low-affinity IL-2 (laIL-2) that has greatly reduced binding to both IL-2Rα and IL-2Rβ. We linked laIL-2 to an anti–PD-1 antibody (generating PD-1–laIL-2) to increase its avidity to intratumoral CD8^+^ T cells. PD-1–laIL-2 showed better intratumoral T cell binding and potent antitumor effects. The use of such fusion proteins can also overcome PD-L1 therapy resistance.

## Results

### PD-1–laIL-2 selectively targets intratumoral CD8^+^ T cells.

**Figure 1:**
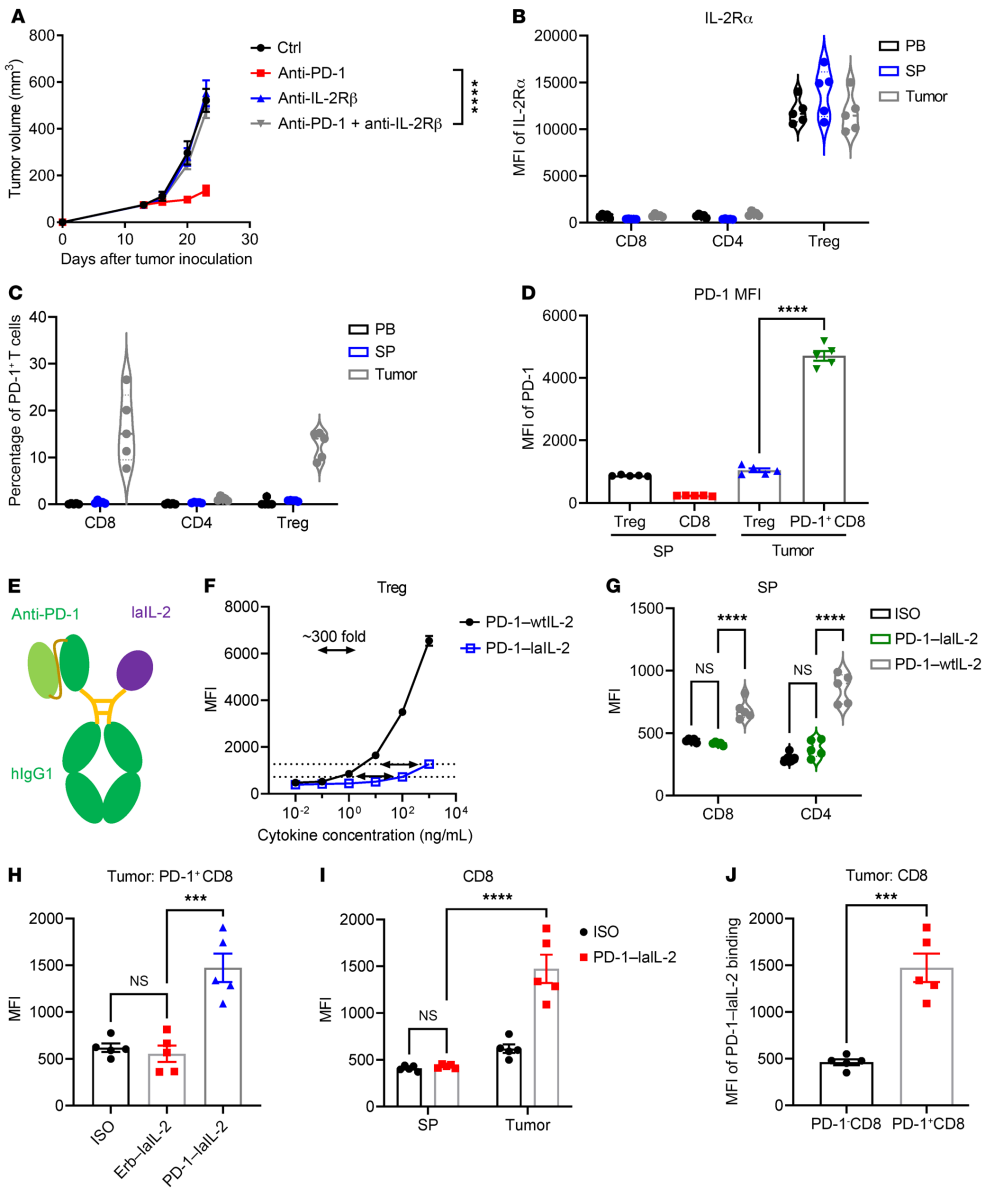
PD-1–laIL-2 selectively targets intratumoral CD8^+^ T cells. (**A**) BALB/c mice (*n =* 5/group) were inoculated with 2 × 10^6^ A20 tumor cells and were treated with 50 μg anti–PD-1 and/or 200 μg anti–IL-2Rβ on day 14. Tumor growth was assessed twice a week. (**B**) IL-2Rα expression on CD8^+^, CD4^+^, and Treg cells in peripheral blood, spleen, and tumor samples (indicated as PB, SP, and tumor in the figures) from A20 tumor-bearing mice (*n =* 5/group). (**C**) Percentages of PD-1^+^ T cells in peripheral blood, spleen, and tumor samples from A20 tumor-bearing mice (*n =* 5/group). (**D**) Mean fluorescence intensity (MFI) of PD-1 on Treg and CD8^+^ T cells in the spleen and on Treg and PD-1^+^CD8^+^ T cells in the tumors from A20 tumor-bearing mice (*n =* 5/group). (**E**) Schematic diagram of the anti–PD-1 × laIL-2 heterodimer (PD-1–laIL-2). (**F** and **G**) PD-1–wtIL-2 and PD-1–laIL-2 bind to Treg (**F**), CD8^+^, and CD4^+^ (**G**) T cells in the spleen of A20 tumor-bearing mice (*n =* 5/group). (**H**) Erb–laIL-2 and PD-1–laIL-2 bind to PD-1^+^CD8^+^ T cells in tumors from A20 tumor-bearing mice (*n =* 5/group). (**I**) PD-1–laIL-2 binds to CD8^+^ T cells in the spleen and to PD-1^+^CD8^+^ T cells in tumors from A20 tumor-bearing mice (*n =* 5/group). (**J**) PD-1–laIL-2 binds to PD-1^–^CD8^+^ and PD-1^+^CD8^+^ T cells in tumors from A20 tumor-bearing mice (*n =* 5/group). Data represent mean ± SEM from 2 to 3 independent experiments. The *P* value was determined by 2-way ANOVA with Geisser-Greenhouse’s correction (**A**), 1-way ANOVA with Tukey’s multiple comparisons test (**D** and **H**), 2-way ANOVA with Tukey’s multiple comparisons test (**G** and **I**), or 2-tailed unpaired *t* test (**J**). The normality of the data was confirmed by the Shapiro-Wilk test. ***P < 0.001, ****P < 0.0001.

To test whether the therapeutic effect of anti–PD-1 depends on IL-2 signaling, we blocked the IL-2 pathway with anti–IL-2Rβ. The therapeutic effect of anti–PD-1 was totally abolished when anti–IL-2Rβ was given (Figure 1A and Supplemental Table 1), which suggests that IL-2 signaling is important for anti–PD-1 immunotherapy. Therefore, we hypothesized that targeting exogenous IL-2 to PD-1^+^ T cells may greatly expand PD-1 blockade-rescued dysfunctional TILs to enhance the therapeutic effect. We examined the expression of IL-2Rα and PD-1 on T cells in tumor-bearing mice. Consistent with previous studies, IL-2Rα was mainly expressed on Treg cells (Figure 1B and Supplemental Figure 1A; supplemental material available online with this article; https://doi.org/10.1172/JCI153604DS1). IL-2Rβ and IL-2Rγ were universally expressed on all T cells (Supplemental Figure 1, B and C). Compared with that in the peripheral blood and spleen, there was a higher percentage of PD-1^+^CD8^+^ T cells in the tumor (Figure 1C). PD-1^+^CD8^+^ T cells expressed higher levels of PD-1 than Treg cells in the tumor (Figure 1, C and D). We proposed PD-1 as an appealing target to bring IL-2 to CD8^+^ TILs. To reduce the binding of IL-2 to Treg cells that express IL-2Rα and IL-2Rβ and potentially absorb more IL-2, we selected a low-affinity IL-2 (IL-2 R38L F42A, laIL-2) that has greatly reduced binding to both IL-2Rα and IL-2Rβ for Treg cells.

Taking advantage of the high expression of PD-1 on CD8^+^ T cells among TILs, we engineered PD-1–laIL-2 to increase their avidity to intratumoral CD8^+^ T cells (Figure 1E and Supplemental Figure 1D). PD-1–laIL-2 had a much lower binding than anti–PD-1–linked wild-type IL-2 (PD-1–wtIL-2) to peripheral Treg cells (Figure 1F and Supplemental Figure 1E). Moreover, the binding of PD-1–laIL-2 to intratumoral Treg cells was much lower than that of PD-1–wtIL-2 (Supplemental Figure 1F). We also checked the binding of PD-1–laIL-2 to HEK cells that express human IL-2 receptors (HEK-Blue IL-2 cells), and the binding of PD-1–laIL-2 to HEK-Blue IL-2 cells was much lower than that of PD-1–wtIL-2 (Supplemental Figure 1, G and H). Compared with PD-1–wtIL-2, PD-1–laIL-2 also had reduced binding to peripheral CD8^+^ and CD4^+^ T cells, which was almost undetectable and should result in reduced toxicity (Figure 1G). To test whether PD-1 is critical for enhanced avidity to PD-1^+^ TILs, we compared PD-1–laIL-2 with control antibody-linked laIL-2 (Erb–laIL-2). Indeed, PD-1–laIL-2 bound much more readily to PD-1^+^CD8^+^ TILs than Erb–laIL-2 (Figure 1H). When comparing TILs and peripheral T cells, PD-1–laIL-2 mainly bound to intratumoral PD-1^+^CD8^+^ T cells but did not bind to peripheral CD8^+^ T cells (Figure 1I). Additionally, the binding of PD-1–laIL-2 to intratumoral PD-1^+^CD8^+^ T cells was much higher than binding to intratumoral PD-1^–^CD8^+^ T cells (Figure 1J). Taken together, these findings indicate that PD-1–laIL-2 can selectively target intratumoral CD8^+^ T cells instead of Treg cells.

### PD-1 antibody–armed laIL-2 has enhanced tumor control.

**Figure 2:**
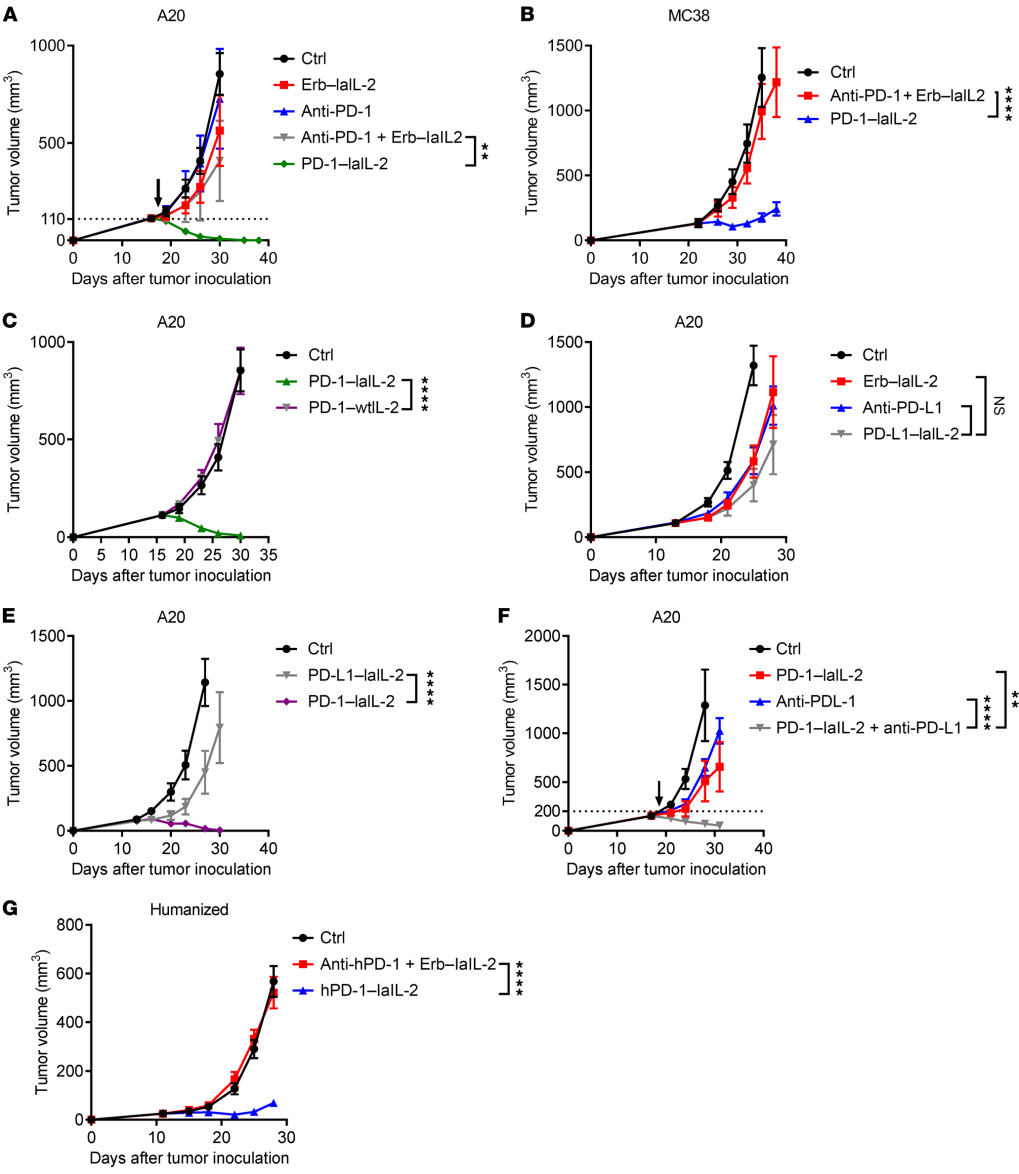
PD-1 antibody–armed laIL-2 has enhanced tumor control. (**A**) A20 tumor-bearing mice (*n =* 5/group) were treated with equal molar amounts of Erb–laIL-2 (20 μg), anti–PD-1 (10 μg), or PD-1–laIL-2 (20 μg) on day 17. Tumor growth was assessed twice a week. (**B**) MC38 tumor-bearing mice (*n =* 5/group) were treated with equal molar amounts of Erb–laIL-2 (20 μg) and anti–PD-1 (10 μg) or PD-1–laIL-2 (20 μg) on day 21. Tumor growth was assessed twice a week. (**C**) A20 tumor-bearing mice (*n =* 5/group) were treated with 20 μg PD-1–laIL-2 or PD-1–wtIL-2 on day 17. Tumor growth was assessed twice a week. (**D**) A20 tumor-bearing mice (*n =* 5/group) were treated with equal molar amounts of Erb–laIL-2 (20 μg), anti–PD-L1 (10 μg), or PD-L1–laIL-2 (20 μg) on day 14. Tumor growth was assessed twice a week. (**E**) A20 tumor-bearing mice (*n =* 5/group) were treated with 20 μg Erb–laIL-2, PD-L1–laIL-2, or PD-1–laIL-2 on day 14. Tumor growth was assessed twice a week. (**F**) A20 tumor-bearing mice (*n =* 5/group) were treated with 20 μg PD-1–laIL-2 and/or 100 μg anti–PD-L1 on day 20. Tumor growth was assessed twice a week. (**G**) A375 tumor-bearing humanized mice (*n =* 5/group) were treated with equal molar amounts of Erb–laIL-2 (20 μg) and anti–hPD-1 (10 μg) or hPD-1–laIL-2 (20 μg) on day 11. Tumor growth was assessed twice a week. Data represent mean ± SEM from 2 to 3 independent experiments. The *P* value was determined by 2-way ANOVA with Geisser-Greenhouse correction (**A**–**G**). **P < 0.01 and ****P < 0.0001.

We then sought to study whether targeting laIL-2 to intratumoral CD8^+^ T cells has a beneficial outcome in terms of tumor control. Strikingly, a single low dose (20 μg) of PD-1–laIL-2 eradicated the established A20 tumors, whereas an equivalent dose of single or combination treatment of anti–PD-1 and Erb–laIL-2 had almost no effect at all (Figure 2A). Importantly, the antitumor effect of PD-1–laIL-2 was not restricted to the A20 tumor model, and PD-1–laIL-2 had much better control of the tumors than anti–PD-1 plus Erb–laIL-2 in the MC38 colon tumor model (Figure 2B) and even the poorly immunogenic Renca renal tumor model (Supplemental Figure 2A). To investigate whether laIL-2 is necessary, we treated tumor-bearing mice with similar molar amounts of PD-1–wtIL-2 and PD-1–laIL-2. PD-1–wtIL-2 had much less tumor control capacity than PD-1–laIL-2 (Figure 2C and Supplemental Figure 2, B and C). These data suggest that reducing the binding of IL-2 to peripheral T cells may allow better tumor targeting. To investigate whether targeting laIL-2 to the tumor site via PD-1 can exert better tumor control than other potential non-T cell targets, we linked laIL-2 to an anti–PD-L1 antibody (PD-L1–laIL-2). PD-L1–laIL-2 had a similar tumor control effect as Erb–laIL-2 or anti–PD-L1 (Figure 2D) and was far less effective than PD-1–laIL-2 (Figure 2E and Supplemental Figure 2C). Therefore, the data suggest that delivering IL-2 to T cells via anti–PD-1 is important. Since PD-1/PD-L1 therapy often fails to control more established tumors, we speculated that the enrichment of IL-2 in TILs can amplify antitumor immunity during anti–PD-1/PD-L1 treatment. To avoid the competition between anti–PD-1 and PD-1–laIL-2 for PD-1 on T cells, we chose anti–PD-L1 treatment. To test whether PD-1–laIL-2 can overcome anti–PD-L1 therapy resistance, we added PD-1–laIL-2 treatment to a PD-L1 therapy regimen and observed that PD-1–laIL-2 could synergize with anti–PD-L1 therapy in more advanced tumors (Figure 2F). Therefore, PD-1–laIL-2 overcomes resistance to PD-L1 blockade therapy. In a humanized mouse model, we observed much better tumor control in the hPD-1–laIL-2 group than in the combination treatment group (Figure 2G).

### Antitumor efficacy of PD-1–laIL-2 depends on intratumoral CD8^+^ T cells.

**Figure 3:**
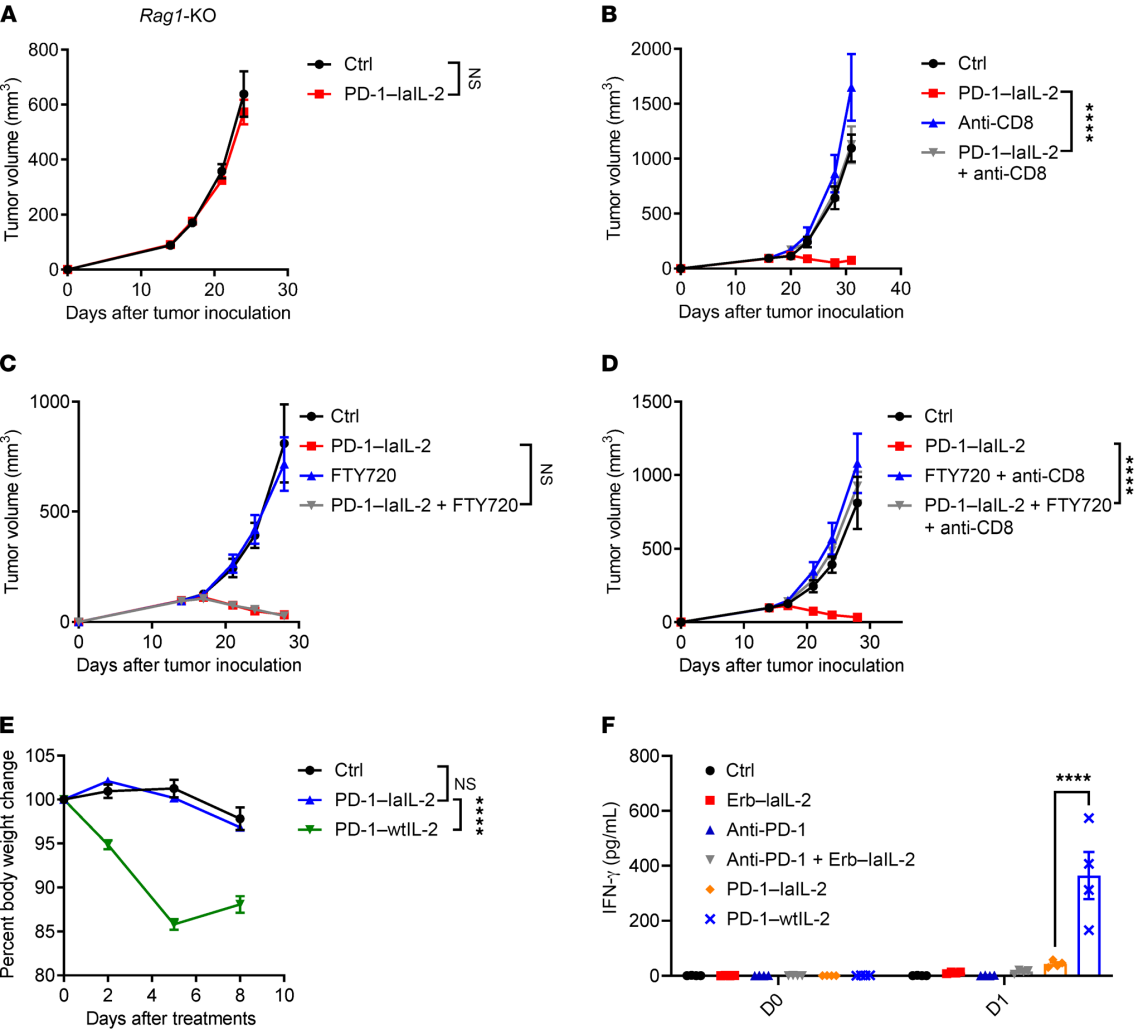
Antitumor efficacy of PD-1–laIL-2 depends on intratumoral CD8^+^ T cells. (**A**) A20 tumor-bearing *Rag1*-KO mice (*n =* 5/group) were treated with 20 μg PD-1–laIL-2 on day 15. Tumor growth was assessed twice a week. (**B**) A20 tumor-bearing mice (*n =* 5/group) were treated with 20 μg PD-1–laIL-2 on day 17. Anti-CD8 (200 μg/mouse) was administered twice a week starting on day 16. Tumor growth was assessed twice a week. (**C**) A20 tumor-bearing mice (*n =* 5/group) were treated with 20 μg PD-1–laIL-2 on day 17. FTY720 was administered every 2 days starting on day 16 through the end of the experiment. Tumor growth was assessed twice a week. (**D**) A20 tumor-bearing mice (*n =* 5/group) were treated with 20 μg PD-1–laIL-2 on day 17. FTY720 was administered every 2 days starting on day 16 through the end of the experiment. Anti-CD8 (200 μg/mouse) was administered twice a week starting on day 16. Tumor growth was assessed twice a week. (**E**) Renca tumor-bearing mice (*n =* 5/group) were treated with 100 μg PD-1–laIL-2 or PD-1–wtIL-2 intraperitoneally. Mouse body weight was monitored twice a week. (**F**) Renca tumor-bearing mice (*n =* 5/group) were treated with equal molar amounts of Erb–laIL-2 (100 μg), anti–PD-1 (50 μg), PD-1–laIL-2 (100 μg), or PD-1–wtIL-2 (100 μg) by intraperitoneal injection. IFN-γ in the serum was measured by cytometric bead array (CBA) one day after the treatment. Data represent mean ± SEM from 2 independent experiments. The *P* value was determined by 2-way ANOVA with Geisser-Greenhouse correction (**A**–**E**) or 2-way ANOVA with Tukey’s multiple comparisons test (**F**). ****P < 0.0001.

As IL-2 can provoke the function of NK and T cells (39), we used *Rag1^–/–^*mice that lack T and B cells but have intact NK cells to dissect the contribution of NK and T cells. The therapeutic effect of PD-1–laIL-2 was totally abolished in *Rag1^–/–^* mice (Figure 3A), which suggests that NK cells are not sufficient for PD-1–laIL-2–induced tumor control and that T cells are required for the therapeutic function of PD-1–laIL-2. NK cell depletion had no impact on tumor control by PD-1–laIL-2 (Supplemental Figure 3A), which further shows that NK cells are not required for PD-1–laIL-2 therapy in the current model. To determine which subset of T cells is required, we depleted either CD4^+^ or CD8^+^ T cells before and during PD-1–laIL-2 treatment. CD4^+^ T cells were not necessary for PD-1–laIL-2 treatment (Supplemental Figure 3B). However, CD8^+^ T cell depletion completely abolished the therapeutic effect of PD-1–laIL-2 (Figure 3B). Therefore, CD8^+^ T cells, but not CD4^+^ T cells or NK cells, are required for the therapeutic effect of PD-1–laIL-2 in our model.

Whether T cells inside the tumor or draining lymph nodes (dLNs) play a more dominant role in anti–PD-1 therapy remains controversial (40, 41). To investigate whether the T cells in the tumor or dLNs are essential for PD-1–laIL-2 treatment in advanced tumors, we used FTY720 to block sphingosine 1–phosphate receptor 1, thereby prohibiting T cells from exiting the lymphoid organs (42). Intriguingly, FTY720 treatment had no impact on the therapeutic effect of PD-1–laIL-2 (Figure 3C), which suggests that T cells from dLNs are not necessary for PD-1–laIL-2 treatment–induced tumor control. Further depletion of CD8^+^ T cells during FTY720 treatment confirmed that CD8^+^ T cells inside the tumor were necessary for PD-1–laIL-2–induced tumor control (Figure 3D). Therefore, for established tumors, TILs might be essential for tumor control after the systemic delivery of fusion proteins.

In the clinic, high-dose IL-2 treatment causes severe side effects (30, 43). To test whether PD-1–laIL-2 treatment also induces side effects, we monitored the body weights of mice during and after the treatment. The body weights of mice were reduced only in the PD-1–wtIL-2–treated group but not in the PD-1–laIL-2–treated group (Figure 3E), which suggests that PD-1–laIL-2 has much lower toxicity than PD-1–wtIL-2. Additionally, there was a much lower level of IFN-γ in the serum of PD-1–laIL-2–treated mice than in that of PD-1–wtIL-2–treated mice (Figure 3F). Together, these data suggest that PD-1–laIL-2 is much safer and more effective than PD-1–wtIL-2.

### PD-1–laIL-2 increases the abundance of tumor-specific CD8^+^ T cells.

**Figure 4:**
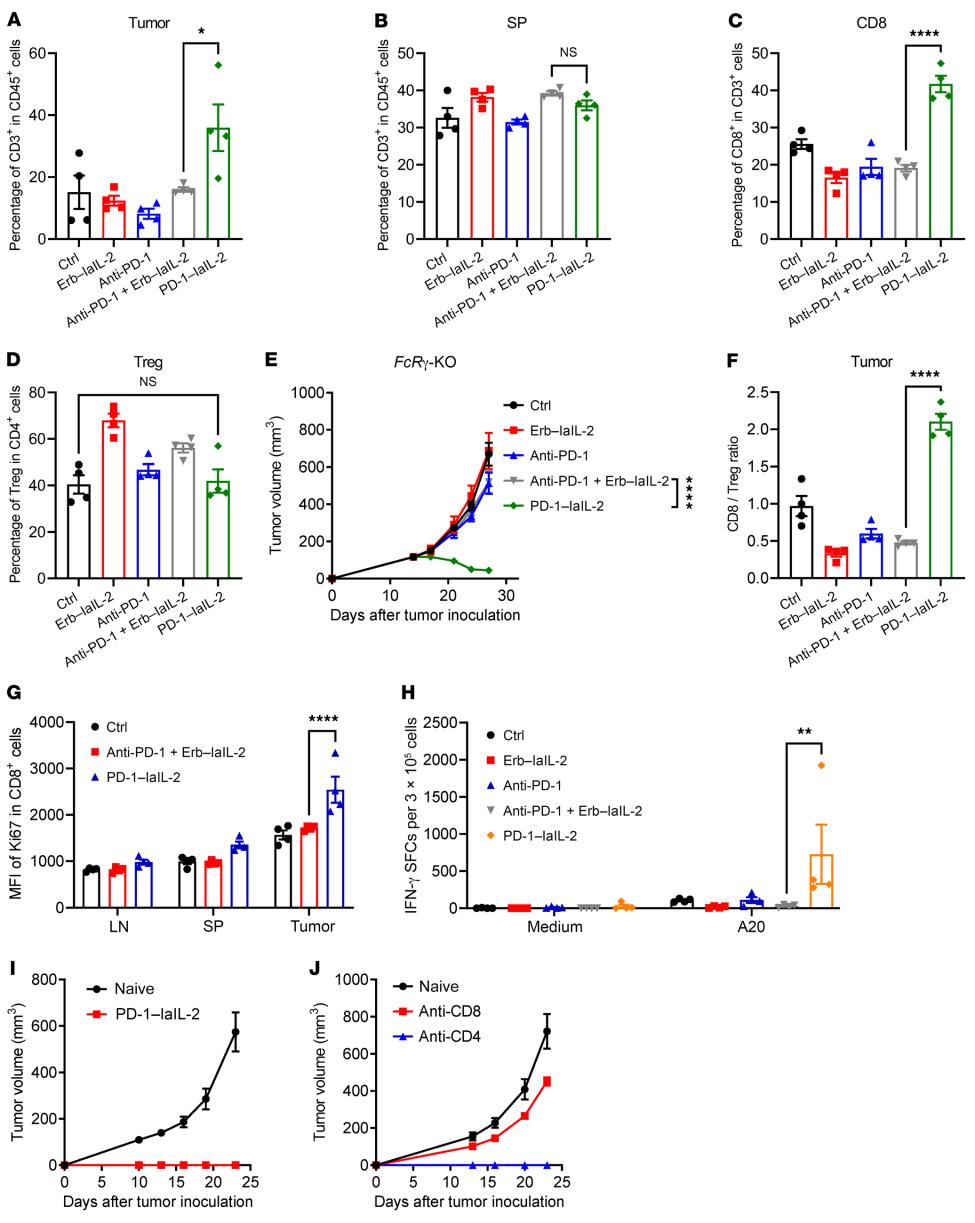
PD-1–laIL-2 increases the abundance of tumor-specific CD8^+^ T cells. (**A**–**D**) A20 tumor-bearing mice (*n* = 5/group) were treated with equal molar amounts of Erb–laIL-2 (20 μg), anti–PD-1 (10 μg) or PD-1–laIL-2 (20 μg) on day 17. Six days later, T cells from the tumor and spleen were analyzed. CD3^+^ T cell frequency in the tumor or spleen from different groups is shown separately in **A** and **B**. CD8^+^ T cell or Treg cell frequencies in tumors from different groups are shown separately in **C** and **D**. (**E**) A20 tumor-bearing *FcR*γ KO mice (*n =* 5/group) were treated with equal molar amounts of Erb–laIL-2 (20 μg), anti–PD-1 (10 μg), or PD-1–laIL-2 (20 μg) on day 15. Tumor growth was assessed twice a week. (**F** and **G**) The mice were treated as in **A**–**D**. Six days later, T cells from the tumor and spleen were analyzed. The ratio of CD8^+^ T cells to Treg cells is shown in **F**. Ki67 expression in CD8^+^ T cells is shown in **G**. (**H**) The mice were treated as in **A**–**F**. Six days later, draining lymph node (dLN) cells were collected, and the IFN-γ ELISPOT assay was performed with medium or irradiated A20 cell restimulation. (**I** and **J**) Approximately 60 days after tumor rejection in PD-1–laIL-2–treated mice (*n =* 10/group), 5 times the original number of A20 cells (1 × 10^7^) were injected on the opposite flank for tumor rechallenge. Anti-CD8 (200 μg/mouse) or anti-CD4 (200 μg/mouse) was administered twice a week starting 1 day before tumor inoculation. Naive WT BALB/c mice (*n =* 5/group) were used as controls. Tumor growth was monitored twice a week. Data represent mean ± SEM from 2 independent experiments. The *P* value was determined by 1-way ANOVA with Tukey’s multiple comparisons test (**A**–**D** and **F**), 2-way ANOVA with Geisser-Greenhouse correction (**E**), or 2-way ANOVA with Tukey’s multiple comparisons test (**G** and **H**). *P < 0.05, **P < 0.01, and ****P < 0.0001.

As T cells are important for PD-1–laIL-2 treatment and PD-1–laIL-2 does not bind to peripheral T cells, we proposed that PD-1–laIL-2 can preferentially expand TILs but not T cells in lymphoid tissues. Indeed, the abundance of T cells in the tumor increased after PD-1–laIL-2 treatment but showed no changes in the combination-treated group (Figure 4A). Interestingly, the T cells in the spleen and dLNs did not increase after PD-1–laIL-2 treatment (Figure 4B and Supplemental Figure 4A). Similarly, PD-1–laIL-2 induced an increase in CD8^+^ TILs but not CD8^+^ T cells in the dLNs or spleen (Figure 4C and Supplemental Figure 4, B and C). This result is consistent with our previous finding that PD-1–laIL-2 could not bind to peripheral T cells (Figure 1I). Treg cells suppress the proliferation and expansion of CD8^+^ T cells, so PD-1–laIL-2 may indirectly induce an increase in CD8^+^ T cells through Treg cell depletion. However, the percentage of Treg cells did not change in the tumor (Figure 4D) but increased in the dLNs and spleen (Supplemental Figure 4, D and E). This might suggest that PD-1–laIL-2 does not induce Treg cell depletion. Since the depletion of Tregs by antibodies depends on the γ subunit of the immunoglobulin Fc receptor (FcRγ), we utilized *FcR*γ knockout (KO) mice to study whether this fusion protein depends on FcRγ for its activity. PD-1–laIL-2 could efficiently control the tumors in *FcR*γ-KO mice (Figure 4E), which further proves that the therapeutic function of PD-1–laIL-2 does not depend on Treg depletion. There was a 4-fold increase in the CD8^+^ T cell to Treg cell ratio in the tumor after PD-1–laIL-2 treatment (Figure 4F), suggesting that PD-1–laIL-2 promotes an immune-active environment in the tumor. Moreover, the ratio of CD8^+^ T cells to Treg cells decreased in the dLNs and spleen (Supplemental Figure 4, F and G), which is consistent with the lack of toxicity observed in PD-1–laIL-2–treated mice. To investigate how the CD8^+^ T cell number increases, we examined Ki67 expression in CD8^+^ T cells. After PD-1–laIL-2 treatment, CD8^+^ T cells expressed much higher Ki67 levels (Figure 4G), which suggests that PD-1–laIL-2 promotes the proliferation of CD8^+^ T cells in the tumor.

To determine whether this fusion protein directly activates and expands tumor-specific T cells, we isolated immune cells from the dLNs and restimulated them with irradiated A20 tumor cells. The IFN-γ ELISPOT assay showed many more immunospots after PD-1–laIL-2 treatment (Figure 4H), which indicates that PD-1–laIL-2 directly increases the tumor-specific T cell response. To test whether PD-1–laIL-2 treatment induces a protective antitumor memory response, we injected 5 times the original number of tumor cells into the opposite site of the treated back of the PD-1–laIL-2–treated mice at approximately 2 months after eliminating the tumors from those mice. All cured mice spontaneously rejected the rechallenged tumors (Figure 4I). However, if the CD8^+^ T cells were depleted before rechallenging, all the mice developed tumors (Figure 4J), indicating the important role of CD8^+^ T cells in protection. CD4^+^ T cells were dispensable for protection (Figure 4J). These data suggest that PD-1–laIL-2 increases the abundance of tumor-specific T cells and induces a potent memory response.

### PD-1–laIL-2 causes the proliferation of PD-1^+^TIM3^+^CD8^+^ effector T cells.

**Figure 5:**
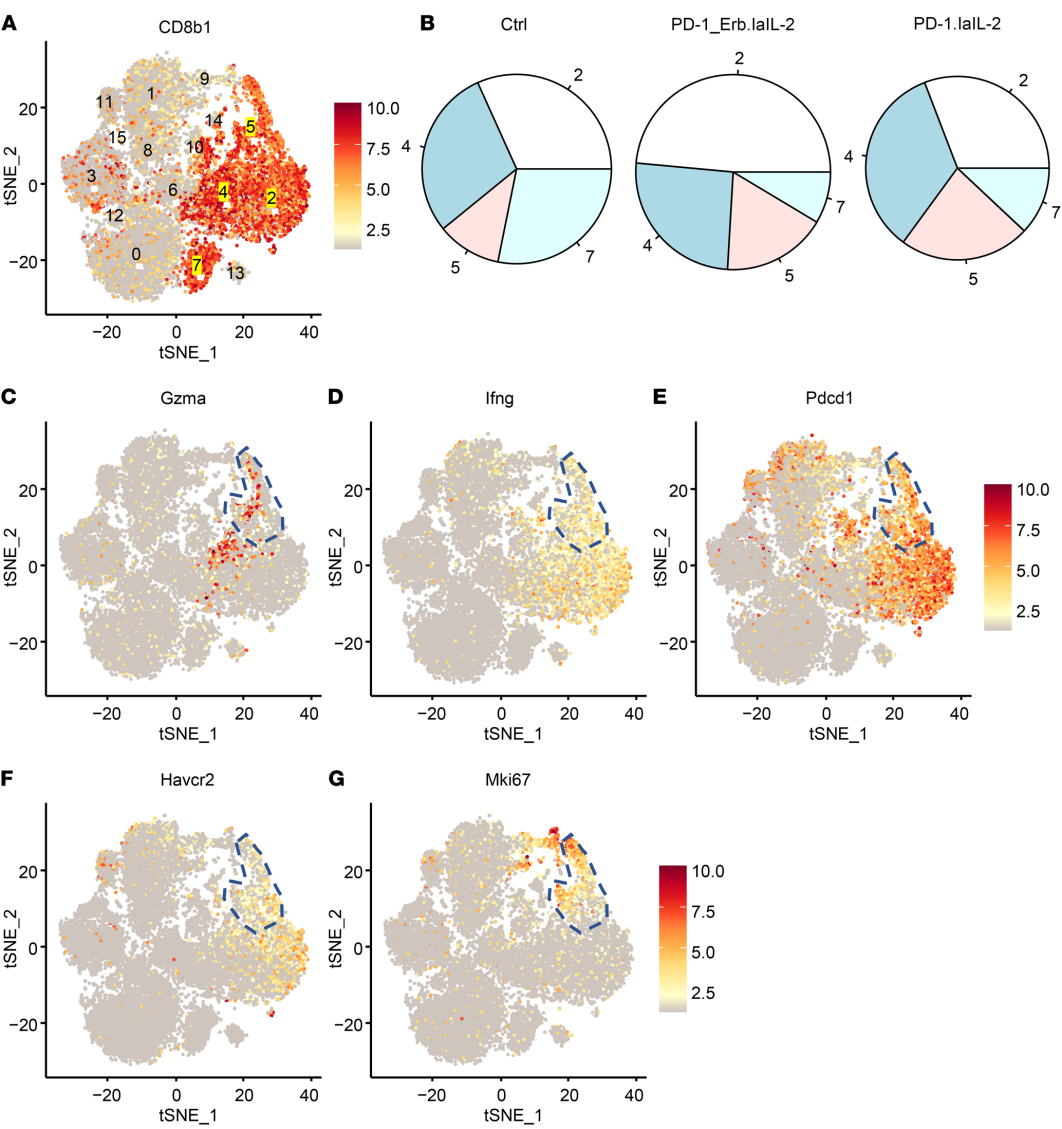
PD-1–laIL-2 causes the proliferation of PD-1^+^TIM3^+^CD8^+^ effector T cells. A20 tumor-bearing mice (*n =* 5/group) were treated with equal molar amounts of Erb–laIL-2 (20 μg) and anti–PD-1 (10 μg) (PD-1_Erb.laIL-2) or PD-1–laIL-2 (20 μg) on day 17. Three days later, CD3^+^ T cells from the tumor were sorted for single-cell RNA sequencing. (**A**) Cd8b1 expression in each cluster. (**B**) Percentages of each cluster in CD8^+^ T cell clusters. (**C**–**G**) Gzma (**C**), IFN-γ (**D**), PD-1 (**E**), TIM3 (**F**), and Ki67 (**G**) expression in each cluster. Cluster 5 was labeled.

To investigate the functional changes in TILs at the single-cell level after PD-1–laIL-2 treatments, we isolated T cells from the tumor for single-cell RNA sequencing. The T cells could be divided into 16 clusters with unsupervised clustering (Supplemental Figure 5A). According to CD8b1 expression, there were 4 CD8^+^ T cell clusters (clusters 2, 4, 5, and 7; Figure 5A), and PD-1–laIL-2 specifically increased the abundance of cluster 5 of CD8^+^ T cells (Figure 5B and Supplemental Figure 5B). Next, we examined the proteins expressed in cluster 5. As expected, cluster 5 had high expression of Gzma (Figure 5C) and IFN-γ (Figure 5D). To our surprise, similar to cluster 2, cluster 5 also expressed high levels of PD-1 (Figure 5E) and TIM3 (Figure 5F). However, cluster 5 also expressed a high level of Ki67 (Figure 5G), making it different from cluster 2. Since PD-1^+^TIM3^+^ T cells are often considered to be dysfunctional and terminally differentiated TILs, our data raise the possibility that PD-1–laIL-2 can promote the proliferation of PD-1^+^TIM3^+^CD8^+^ T cells with effector function.

### PD-1–laIL-2 promotes TIL differentiation into proliferating PD-1^+^TIM3^+^CD8^+^ effector T cells.

**Figure 6:**
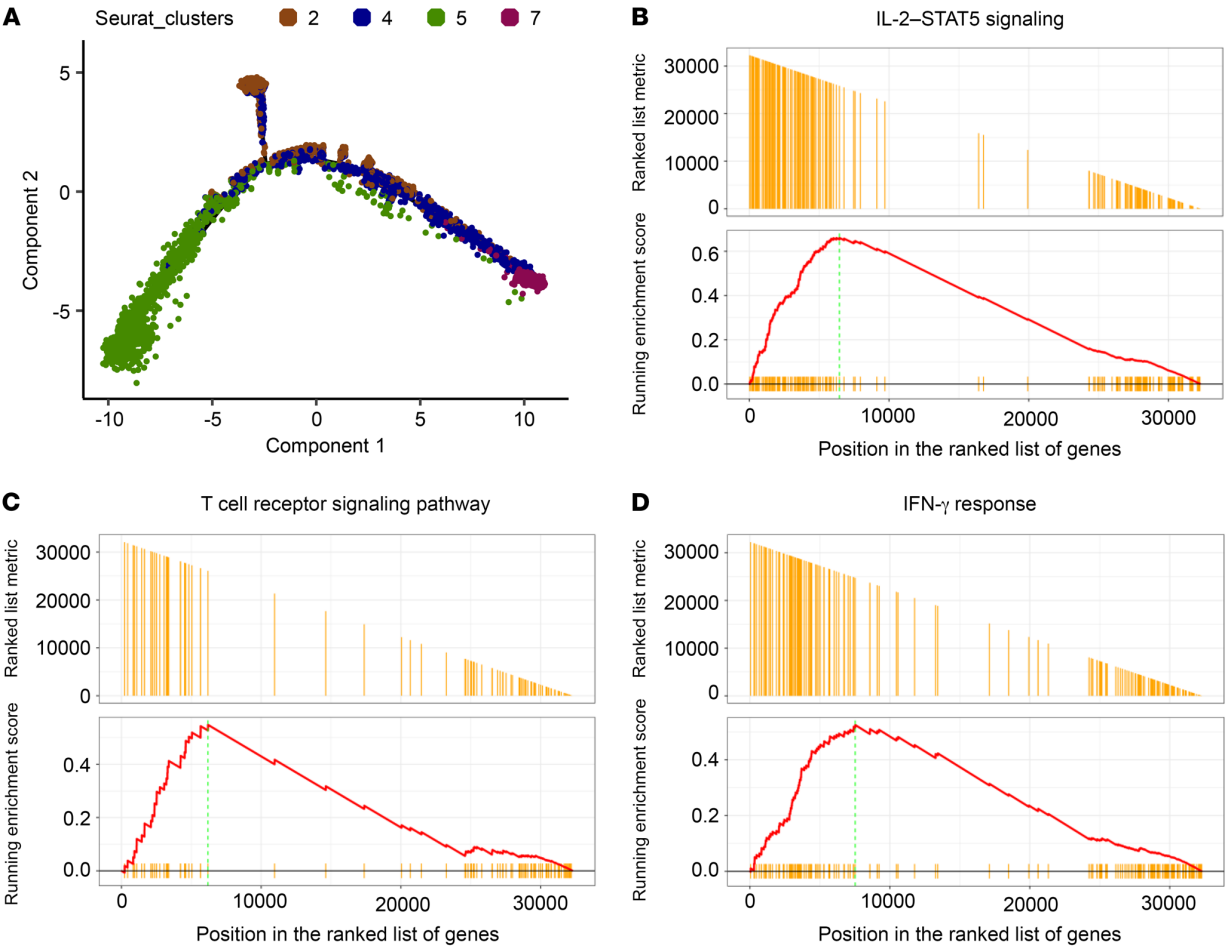
PD-1–laIL-2 promotes TIL differentiation into proliferating PD-1^+^TIM3^+^CD8^+^ effector T cells. A20 tumor-bearing mice (*n* = 5/group) were treated with equal molar amounts of Erb–laIL-2 (20 μg) and anti–PD-1 (10 μg) (PD-1_Erb.laIL-2) or PD-1–laIL-2 (20 μg) on day 17. Three days later, CD3^+^ T cells from the tumor were sorted for single-cell RNA sequencing. (**A**) Single-cell trajectories of CD8^+^ T cell clusters. (**B**–**D**) Gene set enrichment analysis of IL-2–STAT5 signaling (**B**), T cell receptor signaling (**C**), and IFN-γ (**D**) response-related genes in cluster 5 compared with other CD8^+^ T cell clusters.

To test whether PD-1–laIL-2 can promote TIL differentiation into proliferating PD-1^+^TIM3^+^CD8^+^ T cells with effector function, we further analyzed the relationship of CD8^+^ T cell clusters with Monocle. Indeed, T cells in cluster 5 and cluster 2 were all terminally differentiated TILs with distinct phenotypes (Figure 6A and Supplemental Figure 5C), which suggests that PD-1–laIL-2 can promote CD8^+^ T cells to differentiate into proliferating effector TILs. Pathway analysis showed the enrichment of IL-2/STAT5 signaling pathway–related genes (Figure 6B), T cell receptor signaling pathway–related genes (Figure 6C), and IFN-γ response-related genes (Figure 6D) in cluster 5, which indicates that T cells in cluster 5 are enriched tumor-specific T cells and respond more readily to IL-2 stimulation. Taken together, these findings indicate that PD-1–laIL-2 increases the abundance of proliferating PD-1^+^TIM3^+^CD8^+^ effector T cells.

### PD-1–laIL-2 specifically reactivates PD-1^+^TIM3^+^ tumor-specific CD8^+^ T cells.

**Figure 7:**
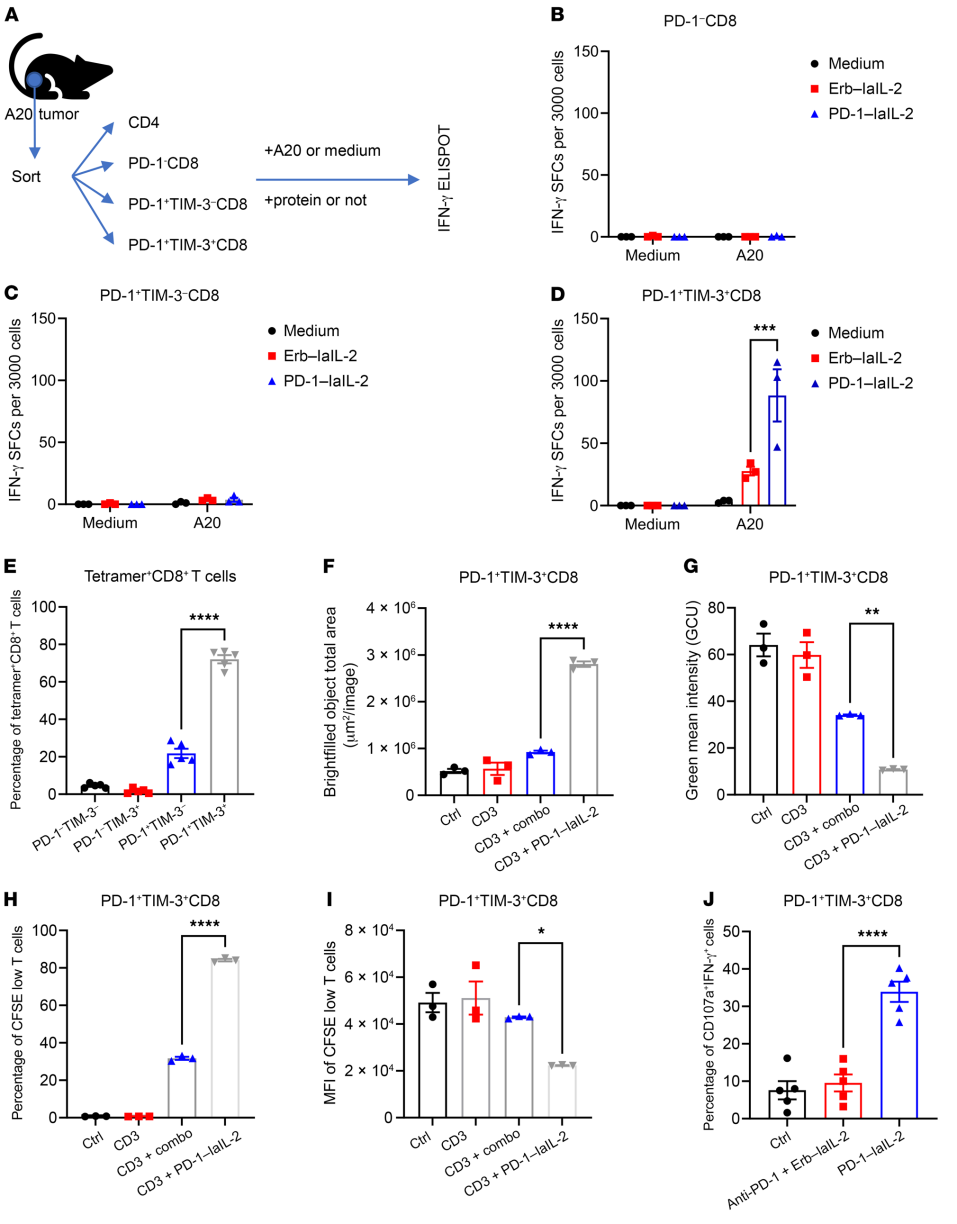
PD-1–laIL-2 specifically reactivates PD-1^+^TIM3^+^ tumor-specific CD8^+^ T cells. (**A**–**D**) CD4^+^, PD-1^–^CD8^+^, PD-1^+^TIM3^–^CD8^+^, and PD-1^+^TIM3^+^CD8^+^ T cells from A20 tumor-bearing mice were sorted out and cocultured with irradiated A20 cells in the presence of Erb–laIL-2 or PD-1–laIL-2 for the IFN-γ ELISPOT assay. Experimental scheme (**A**) and spots from PD-1^–^CD8^+^ (**B**), PD-1^+^TIM3^–^CD8^+^ (**C**) and PD-1^+^TIM3^+^CD8^+^ (**D**) T cells are shown. (**E**) PD-1 and TIM3 expressions on tetramer^+^CD8^+^ T cells in tumors from MC38 tumor-bearing mice (*n =* 5). (**F**–**I**) Splenocytes were stimulated with anti-CD3 and anti-CD28 antibodies. Five days later, PD-1^+^TIM3^+^CD8^+^ T cells were sorted out and labeled with CFSE. Then, the cells were cultured in 96-well plates in the presence of anti-CD3, Erb–laIL-2 plus anti–PD-1 (combo), or PD-1–laIL-2 for 2 days. The T cell clusters and CFSE expression were assayed with an Incucyte instrument. Total areas of the cluster are shown in **F**. Mean CFSE intensity is shown in **G**. (**H**) Percentage of CFSE low cells. (**I**) MFI of CFSE low cells. (**J**) A20 tumor-bearing mice (*n =* 5/group) were treated with equal molar amounts of Erb–laIL-2 (20 μg), anti–PD-1 (10 μg), or PD-1–laIL-2 (20 μg) on day 19. Six days later, T cells from the tumor were analyzed. Data represent mean ± SEM. The *P* value was determined by 2-way ANOVA with Tukey’s multiple comparisons test (**D**) or 1-way ANOVA with Tukey’s multiple comparisons test (**E**–**J**). *P < 0.05, **P < 0.01, ***P < 0.001, and ****P < 0.0001.

To confirm whether PD-1–laIL-2 can increase the effector function of some subsets of TILs, we sorted CD4^+^, PD-1^–^CD8^+^, PD-1^+^TIM3^–^CD8^+^, and PD-1^+^TIM3^+^CD8^+^ T cells from the tumor and treated them with PD-1–laIL-2 in the presence of irradiated A20 tumor cells (Figure 7A). It is widely believed that PD-1^–^ TILs might be newly arrived cells, PD-1^+^TIM3^–^ TILs are newly activated effector cells and PD-1^+^TIM3^+^ TILs are exhausted cells (44, 45). To our surprise, PD-1–laIL-2 could not induce IFN-γ expression in CD4^+^, PD-1^–^CD8^+^ or PD-1^+^TIM3^–^CD8^+^ T cells (Figure 7, B and C, and Supplemental Figure 6A). However, IFN-γ–producing cells stimulated by tumors were primarily enriched inside the population of PD-1^+^TIM3^+^CD8^+^ T cells after PD-1–laIL-2 treatment (Figure 7D). These data suggest that PD-1–laIL-2 could increase the function of PD-1^+^TIM3^+^CD8^+^ T cells. Furthermore, these results also indicate that PD-1^+^TIM3^+^ cells are the tumor-specific T cells in the tumor. To directly check the expression of PD-1 and TIM3 on tumor-specific T cells, we used tetramers to track tumor-specific T cells in the MC38 tumor model. The results showed that most of the tetramer-positive CD8^+^ T cells were PD-1 and TIM3 double positive (Figure 7E and Supplemental Figure 6B). Therefore, most tumor-specific T cells responding to IL-2 are PD-1^+^TIM3^+^ cells.

To investigate whether PD-1–laIL-2 can directly promote the proliferation of PD-1^+^TIM3^+^CD8^+^ T cells, we labeled PD-1^+^TIM3^+^CD8^+^ T cells isolated from anti-CD3– and anti-CD28–treated splenocytes with carboxyfluorescein succinimidyl ester (CFSE) and treated them with PD-1–laIL-2 or the combination of anti–PD-1 and Erb–laIL-2. Two days later, we examined the morphological phenotypes and CFSE density of these T cells with an Incucyte system. PD-1–laIL-2 greatly increased the size of the T cell clusters (Figure 7F and Supplemental Figure 6C), which suggests that PD-1–laIL-2 increases the total number of T cells. PD-1–laIL-2 also reduced the mean green intensity (Figure 7G and Supplemental Figure 6C), indicating the dilution of CFSE after cell division. We also confirmed the reduction in CFSE brightness by flow cytometry (Figure 7, H and I, and Supplemental Figure 6D). To examine whether PD-1–laIL-2 can effectively recover the functionality of dysfunctional T cells, we examined the cytotoxic function of PD-1^+^TIM3^+^ CD8^+^ T cells from the tumor-bearing mice after PD-1–laIL-2 treatment. Indeed, PD-1–laIL-2 increased the frequency of CD107a^+^IFN-γ^+^ cells among PD-1^+^TIM3^+^CD8^+^ T cells in the TME (Figure 7J). Therefore, PD-1–laIL-2 can reactivate PD-1^+^TIM3^+^ tumor-specific CD8^+^ T cells in the tumor.

## Discussion

Immunogenic tumor tissues often have a high number of T cells, but their dysfunction limits their capacity to control tumors (13, 46). PD-1^+^ and TIM3^+^ TILs are considered to be terminally differentiated and dysfunctional TILs (44). Anti–PD-1/PD-L1 treatment can release the brake on the T cell response and partially restore their functions, but only a small number of patients have complete responses (9). Unexpectedly, we observed that IL-2 is required for optimal PD-1 therapy. This raises the possibility that targeting TILs with IL-2 might overcome PD-1 resistance. However, due to the high expression of IL-2 receptors on Treg cells, it is difficult to deliver enough IL-2 to CD8^+^ T cells in the tumor while avoiding systemic toxicity. Therefore, we designed a PD-1–laIL-2 fusion protein by linking low-affinity IL-2 to an anti–PD-1 antibody to target intratumoral PD-1–high CD8^+^ T cells instead of Tregs. PD-1–laIL-2 had much lower binding to peripheral CD8^+^ T cells and Treg cells, which greatly reduced peripheral consumption. A single low-dose of PD-1–laIL-2 treatment eradicated the tumors. PD-1–laIL-2 treatment favors CD8^+^ T cells over Treg cells in the tumor. Intriguingly, PD-1–laIL-2 could reactivate PD-1^+^TIM3^+^CD8^+^ TILs, and a long-term memory response was generated to protect against relapse. Overall, PD-1–laIL-2 is a next-generation PD-1 therapy that can target tumor-specific T cells.

One major obstacle for IL-2 cancer immunotherapy is the high expression of IL-2Rα on Treg cells (30, 37). Therefore, high-dose IL-2 must be used to increase the accessibility of CD8^+^ T cells to IL-2 in the clinic, which in turn increases severe toxicities due to strong CD8^+^ T cell activation (39). To make IL-2 selectively bind to CD8^+^ T cells, our group and others have tried to reduce the binding of IL-2 to Treg cells (28, 35). The F42A mutation was used to reduce the binding of IL-2 to IL-2Rα. While this mutation reduces the binding to Tregs, it still activates circulating CD8^+^ T cells and increases cytokine release in the periphery (28). The laIL-2 we used in this study maintains the low binding feature to IL-2Rα and further reduces binding to IL-2Rβ (47). As a result, laIL-2 cannot activate peripheral CD8^+^ T cells or Treg cells but is able to activate CD8^+^ T cells in the tumor when paired with anti–PD-1. This is critical in reducing IL-2 treatment–induced systemic toxicity. During the revision of our manuscript from a preprint website (Research Square), we noticed that Roche is also testing a similar fusion protein. The IL-2 variant that they use is only devoid of IL-2Rα binding. This is different from the IL-2 we use in the present study, as we also reduce the IL-2 binding to IL-2Rβ, which will not induce systemic toxicity.

Most of the studies focus on targeting T cell–driven cytokines to tumor cells or tumor stromal cells (32, 34, 36, 37). This will increase cytokine retention in tumor tissues, but the accessibility of effector T cells to those cytokines might still be limited. It is unclear whether tumor cells or stromal cells are the most desirable targets for T cell–associated cytokines, as some tumor cells are not in contact with T cells, and the internalization of cytokines by tumor cells might reduce the accessibility of TILs to cytokines. Current studies suggest that targeting cytokines to intratumoral effector T cells, preferentially CD8^+^ TILs, might be an efficient yet challenging approach to cancer immunotherapy. The ideal design is targeting cytokines to tumor-specific T cells. However, to the best of our knowledge, no unique markers of tumor-specific T cells have been identified and utilized. An alternative candidate molecule may have the following features: (a) high expression on intratumoral CD8^+^ T cells, (b) functional involvement in antigen-driven T cell activation, and (c) a defined cell subtype that is sensitive to cytokine accessibility. Previous studies have shown that PD-1 is highly expressed on antigen-experienced T cells (48). These cells are the principal subsets responding to immunotherapy and might rely on additional cytokine signaling for rapid antigen-driven proliferation. These features make PD-1 an intriguing target for delivering IL-2 to antigen-specific T cells in the tumor. Indeed, a previous study suggested that combining PD-L1 blockade with IL-2 administration augments T cell responses under conditions of chronic infection (49). This strategy might not be applied to cancer immunotherapy because tumor microenvironments are heavily infiltrated with immunosuppressive Treg cells that are more likely to benefit from IL-2 (50, 51). Our PD-1–laIL-2 specifically targets laIL-2 to antigen-specific intratumoral CD8^+^ T cells and benefits effector T cells instead of Treg cells.

The insufficiency of IL-2 may be a critical issue in T cell exhaustion development. Interestingly, tumor-specific T cells are enriched in PD-1^+^ IM3^+^ TILs, an assumed exhausted T cell population. Indeed, our PD-1–laIL-2 specifically delivers IL-2 to exhausted tumor-specific T cells marked by high PD-1 and TIM3 expression and expands them with enhanced antitumor polyfunctionality, as indicated by high expression of IFN-γ and CD107a. This implies that IL-2 signaling might potentially antagonize PD-1 signaling to maintain the polyfunctionality of antigen-specific T cells. The precise mechanism is unclear and needs further investigation. Low-dose PD-1–laIL-2 effectively rejuvenates the effector function of PD-1^+^TIM3^+^ TILs. This also suggests that PD-1^+^TIM3^+^ TILs are more sensitive to IL-2 than other populations, such as Tregs, and reinforces our rationale that targeted delivery of low amounts of IL-2 to antigen-specific T cells enhances antitumor efficacy without causing side effects.

Overall, our data demonstrate that our next-generation PD-1–laIL-2 construct can carry the T cell growth factor IL-2 to tumor-specific T cells in a safe and effective manner. Our study also revealed a new target for tumor-specific T cells and the novel function of IL-2 in reactivating PD-1^+^TIM3^+^ T cells.

## Methods

### Mice.

Eight-week-old WT BALB/c and C57BL/6 mice were purchased from The Jackson Laboratory. *Rag1^–/–^* (*Rag1*^tm1Mom^/J) and NOD scid gamma mice were maintained internally. All mice were maintained under specific pathogen–free conditions. Animal care and experiments were carried out under institutional and National Institutes of Health protocols and guidelines.

### Cell lines and reagents.

A20 and Renca cells were purchased from the American Type Tissue Culture Collection (ATCC). MC38 cells were purchased from Kerafast. All cell lines were routinely tested using a mycoplasma contamination kit (R&D Systems). A20 and Renca cells were cultured in RPMI 1640 medium supplemented with 10% heat-inactivated fetal bovine serum, 2 mmol/L l-glutamine, 0.1 mmol/L MEM nonessential amino acids, 100 U/mL penicillin, and 100 U/mL streptomycin under 5% CO_2_ at 37°C. MC38 cells were cultured in Dulbecco’s modified Eagle’s medium supplemented with 10% heat-inactivated fetal bovine serum, 100 U/mL penicillin, and 100 U/mL streptomycin under 5% CO_2_ at 37°C.

Anti–IL-2Rβ (clone TM-β1), anti-CD4 (clone GK1.5), anti-CD8 (clone 53–5.8), and anti–PD-1 (clone J43) were purchased from Bio X Cell. Anti–PD-L1 (atezolizumab) was provided by the UT Southwestern Simmons Cancer Center Pharmacy. FTY720 (fingolimod) was purchased from Selleck Chemicals. PD-1–laIL-2, PD-1–wtIL-2, Erb–laIL-2, Erb–wtIL-2, and PD-L1–laIL-2 were produced in house by linking IL-2 to antibodies. Briefly, single-chain variable fragment of antibodies with Fc was cloned into pEE6.4 (Lonza). IL-2 was fused with Fc with a GGGGS linker and cloned into pEE6.4. Heterodimerization of Ab–IL-2 was achieved by the knobs-into-holes approach (52). Plasmids encoding Ab-Fc and IL-2–Fc were cotransfected into FreeStyle 293-F cells (Thermo Fisher Scientific). The supernatant containing fusion proteins was purified with Protein A affinity chromatography (Repligen) according to the manufacturer’s instructions.

### Tumor growth and treatment.

A20 cells (2–10 × 10^6^), Renca cells (4 × 10^5^), or MC38 cells (1 × 10^6^) were injected subcutaneously into the backs of 8- to 10-week-old mice. The tumor volumes were measured along 3 orthogonal axes (a, b, and c) and were calculated as follows: tumor volume = abc/2. The mice were treated with intraperitoneal injections of 20 μg of PD-1–laIL-2, PD-1–wtIL-2, Erb–laIL-2, Erb–wtIL-2, or PD-L1–laIL-2. For CD8^+^ T cell or CD4^+^ T cell depletion experiments, 200 μg anti-CD8 antibody or 200 μg anti-CD4 antibody was injected intraperitoneally 1 day before PD-1–laIL-2 treatment. Two hundred micrograms of anti–IL-2Rβ antibody were used for IL-2 pathway blockade.

### Flow cytometry.

Single-cell suspensions of cells were incubated with anti-CD16/32 (anti-FcγIII/II receptor, clone 2.4G2) for 30 minutes and then stained with conjugated antibodies. All fluorescently labeled antibodies were purchased from BioLegend or BD Bioscience. Samples were analyzed on a CytoFLEX ﬂow cytometer (Beckman Coulter Inc.), and data were analyzed using FlowJo software (TreeStar).

### T cell isolation.

Tumor tissues were excised and digested with 1 mg/mL collagenase A (Roche) and 0.5 mg/mL DNase I (Roche) at 37°C for 30 minutes and then passed through a 70-μm cell strainer to remove large pieces of undigested tumor fragments. CD90^+^ cells were isolated with EasySep Mouse CD90.2 Positive Selection Kit II (STEMCELL Technologies Inc.) according to the manufacturer’s instructions. CD90^+^ cells were then stained with surface marker antibodies. CD4^+^, PD-1^–^CD8^+^, PD-1^+^TIM3^–^CD8^+^, and PD-1^+^TIM3^+^CD8^+^ T cells were sorted by BD FACSAria III (BD Biosciences).

### Measurement of IFN-γ–secreting T cells by ELISPOT assay.

dLN cells were resuspended in RPMI 1640 medium supplemented with 10% FBS, 2 mmol/L l-glutamine, 100 U/mL penicillin, and 100 mg/mL streptomycin. A total of 3 × 10^5^ dLN cells was used for the assay. A20 cells were irradiated with a single dose of 60 Gy. The ratio of A20 to immune cells was 1:4. After 48 hours of incubation, IFN-γ production was determined with an IFN-γ ELISPOT Assay Kit (BD Biosciences) according to the manufacturer’s protocol. The visualized cytokine spots were enumerated with an ImmunoSpot S6 Analyzer (Cellular Technology Limited).

### Single-cell RNA sequencing.

Single-cell suspensions were loaded onto a Chromium Single Cell Chip (10x Genomics) according to the manufacturer’s instructions for coencapsulation with barcoded gel beads at a target capture rate of approximately 10,000 individual cells per sample. Captured mRNA was barcoded during cDNA synthesis, and the barcoded cDNA was converted into pooled single-cell RNA-seq libraries for Illumina sequencing by using the Chromium Single Cell 5′ Library & Gel Bead Kit (10x Genomics) according to the manufacturer’s instructions. All samples were processed simultaneously with the Chromium Controller (10x Genomics) and the resulting libraries were prepared in parallel in a single batch. All libraries were sequenced with a NovaSeq 6000 sequencing system. Demultiplexing of sequencing results, barcode processing, read alignment, and unique molecular identifier (UMI) counting were performed using the 10x Cell Ranger analysis pipeline v6.0. Further quality control, feature selection, dimension reduction, unsupervised clustering, and differential expression analyses were performed using the Seurat R package v4.1.1 (53). Data are available at the Zenodo database (https://doi.org/10.5281/zenodo.5544128).

### In vitro splenocyte activation.

Splenocytes (3 × 10^6^/mL) were activated with anti-CD3 (BioLegend, 1 μg/mL) and anti-CD28 (BioLegend, 1 μg/mL) antibodies for 5 days. The cells were then subjected to surface marker staining and sorted with a BD FACSAria III (BD Biosciences).

### Statistics.

Data are shown as the mean ± SEM. Statistical analyses were performed using a 2-tailed unpaired Student’s *t* test and Prism software (version 9.1, GraphPad Software). Two-way ANOVA was used to compare continuous outcomes across multiple experimental groups.

### Study approval.

This study was approved by the Institutional Animal Care and Use Committee of the University of Texas Southwestern Medical Center (animal protocol number [APN] 2015-101350, APN 2018-102474). 

## Author contributions

ZR, AZ, and YXF designed experiments, analyzed data, and wrote the manuscript. ZR and AZ developed the study methodology. ZR, AZ, ZS, YL, and JY performed experiments. JQ and BL provided reagents. ZR and YXF supervised the project.

## Supplementary Material

Supplemental data

## Version 1 02/01/2022

Electronic publication
