# Supplementary material for: Selective delivery of low-affinity IL-2 to PD-1+ T cells rejuvenates antitumor immunity with reduced toxicity
Source: J Clin Invest. 2022 Feb 1;132(3):e153604. doi: 10.1172/JCI153604 (PMC8803347; doi:10.1172/JCI153604)
Supplement: Supplemental data [file jci-132-153604-s203.pdf]

Supplemental material

Supplemental Table. Used antibody list

Antibody	Company	Catalog number
InVivoMAb anti-mouse CD4 (GK1.5)	Bio X Cell	Cat# BE0003-1
InVivoMAb anti-mouse CD8 (53-5.8)	Bio X Cell	Cat# BE0223
InVivoMAb anti-mouse IL2Rβ (TM-Beta 1)	Bio X Cell	Cat# BE0298
InVivoMAb anti-mouse PD1 (J43)	Bio X Cell	Cat# BE0033-2
Anti asialo GM1 (Rabbit) EX	Wako	Cat# 986-10001
Anti-PDL1 (Atezolizumab)	Genentech	TECENTRIQ®
Ultra-LEAF™ Purified anti-mouse CD3ε	BioLegend	Cat# 100340
Ultra-LEAF™ Purified anti-mouse CD28	BioLegend	Cat# 102116
Anti-mCD45 (Flow cytometry, 30-F11)	BioLegend	Cat# 103126
Anti-mCD3 (Flow cytometry, 145-2C11)	BD Biosciences	Cat# 564379
Anti-mCD4(Flow cytometry, RM4-5)	BioLegend	Cat# 100526
Anti-mCD8 (Flow cytometry, 53-6.7)	BioLegend	Cat# 100730
Anti-mFoxp3 (Flow cytometry, MF-14)	BioLegend	Cat# 126408
Anti-mKi67 (Flow cytometry, 16A8)	BioLegend	Cat# 652413
Anti-mPD1 (Flow cytometry, 29F.1A12)	BioLegend	Cat# 135224
Anti-mTIM3 (Flow cytometry, RMT3-23)	BioLegend	Cat# 119716
Anti-mCD107a (Flow cytometry, 1D4B)	BioLegend	Cat# 121608
Anti-mIFNγ (Flow cytometry, XMG1.2)	BioLegend	Cat# 505808
Anti-mIL2Rα (Flow cytometry, PC61)	BD Biosciences	Cat# 564021

Anti-mIL2Rβ (Flow cytometry, 5H4)	BD Biosciences	Cat# 744995
Anti-mIL2Rγ (Flow cytometry, 4G3)	BD Biosciences	Cat# 554457
Anti-FcγIII/II receptor (clone 2.4G2)	BD Biosciences	Cat# 553141

Supplemental Figure 1. PD1-laIL2 selectively targets intratumoral CD8⁺ T cells. (Related to Figure 1)

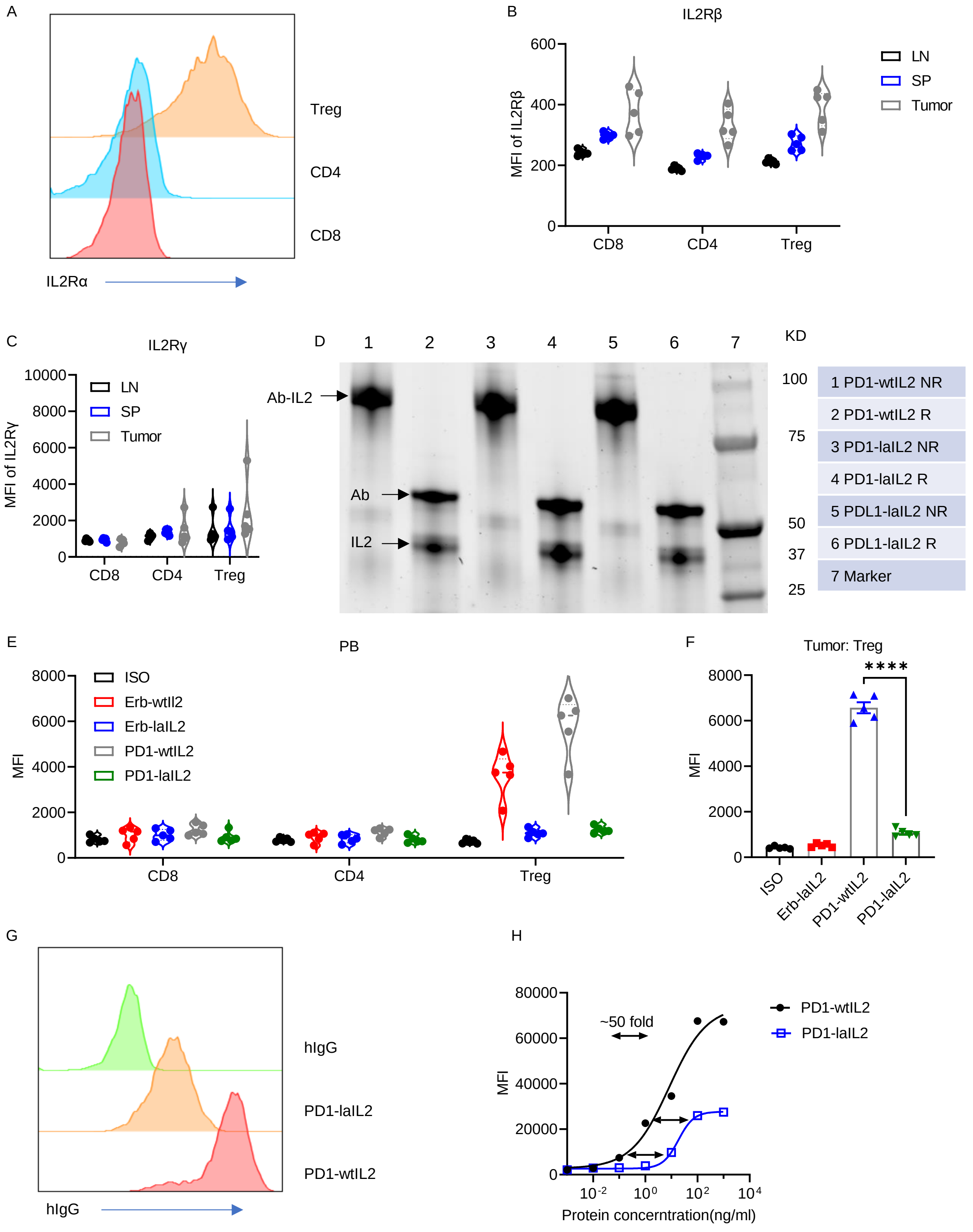

(**A**) Representative IL2Rα expression on $CD8^+$, $CD4^+$ and Treg cells in the spleens of A20 tumor-bearing mice.

(**B, C**) IL2Rβ (**B**) and IL2Rγ (**C**) expression on $CD8^+$, $CD4^+$ and Treg cells in lymph node, spleen and tumor samples (indicated as LN, SP and tumor in the figures) from tumor-bearing mice (n = 5/group).

(**D**) SDS-PAGE of purified proteins under nonreducing (NR) and reducing (R) conditions.

(**E**) Erb-wtIL2, Erb-laIL2, PD1-wtIL2 and PD1-laIL2 bind to Treg, $CD8^+$ and $CD4^+$ T cells in the peripheral blood (PB) of A20 tumor-bearing mice (n = 5/group).

(**F**) Erb-laIL2, PD1-wtIL2 and PD1-laIL2 bind to Treg cells in the tumor of tumor-bearing mice (n = 5/group).

(**G, H**) PD1-wtIL2 and PD1-laIL2 bind to HEK-Blue™ IL-2 cells.

Data represent mean ± SEM. The *P* value was determined by one-way ANOVA with Tukey's multiple comparisons test (**F**).

Supplemental Figure 2. PD1 antibody-armed laIL2 has enhanced tumor control. (Related to Figure 2)

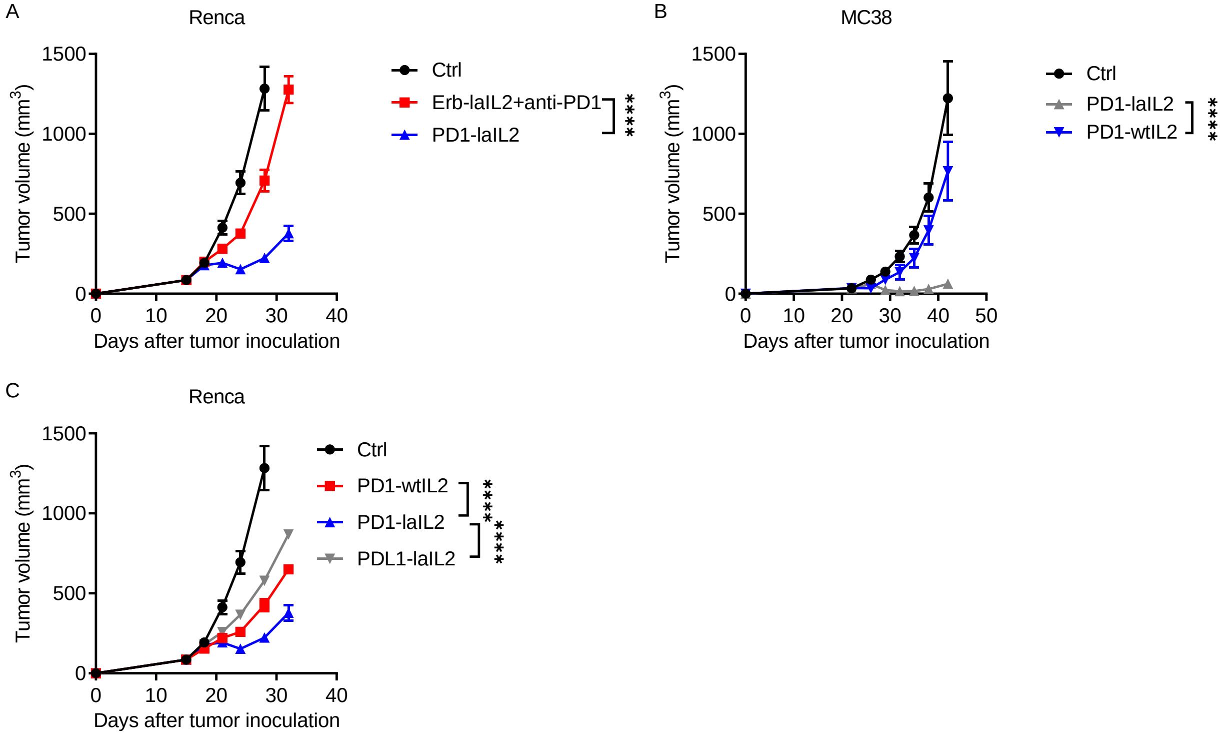

(**A**) Renca tumor-bearing mice (n = 5/group) were treated with equal molar amounts of Erb-laIL2 (100 µg) and anti-PD1 (50 µg) or PD1-laIL2 (100 µg) on day 16. Tumor growth was assessed twice a week.

(**B**) MC38 tumor-bearing mice (n = 5/group) were treated with 20 µg of PD1-laIL2 or PD1-wtIL2 on day 22. Tumor growth was assessed twice a week.

(**C**) Renca tumor-bearing mice (n = 5/group) were treated with 100 µg of PD1-laIL2, PD1-wtIL2, or PDL1-laIL2 on day 16. Tumor growth was assessed twice a week.

Data represent mean ± SEM. The *P* value was determined by two-way ANOVA with Geisser-Greenhouse correction (**A-C**).

Supplemental Figure 3. Antitumor efficacy of PD1-laIL2 depends on intratumoral CD8⁺ T cells. (Related to Figure 3)

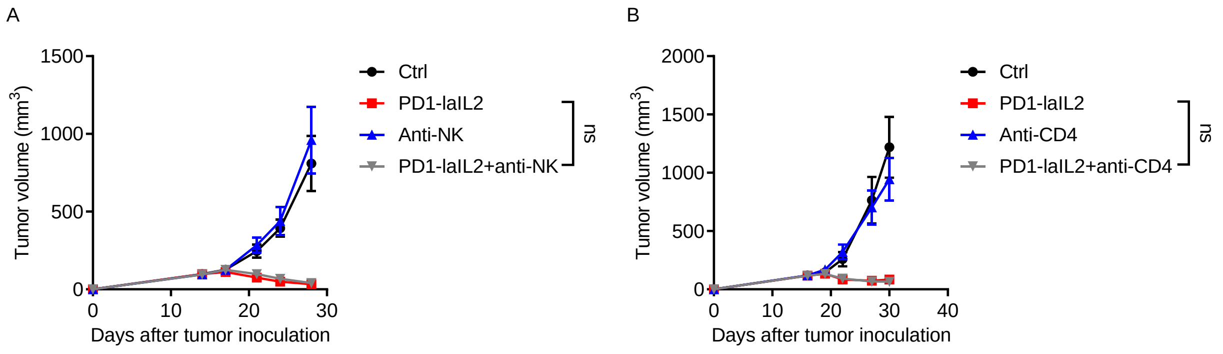

(**A**) A20 tumor-bearing mice (n = 5/group) were treated with 20 µg of PD1-laIL2 on day 17. Anti-NK antibody (20 µg/mouse) was administered twice a week starting on day 16. Tumor growth was assessed twice a week.

(**B**) A20 tumor-bearing mice (n = 5/group) were treated with 20 µg of PD1-laIL2 on day 17. Anti-CD4 (200 µg/mouse) was administered twice a week starting on day 16. Tumor growth was assessed twice a week.

Data represent mean ± SEM. The *P* value was determined by two-way ANOVA with Geisser-Greenhouse correction (**A, B**).

Supplemental Figure 4. PD1-laIL2 increases the abundance of tumor-specific CD8⁺ T cells. (Related to Figure 4)

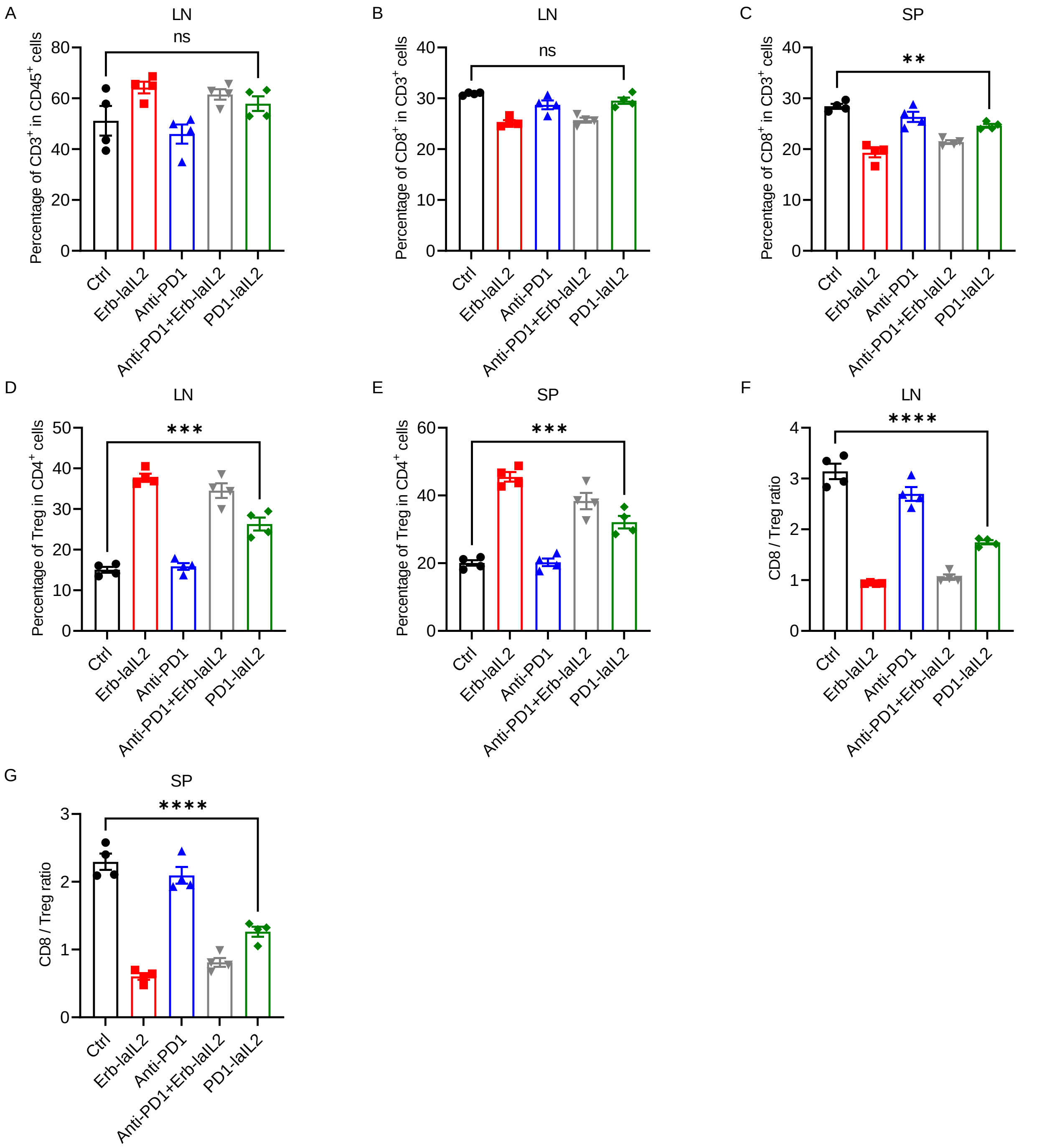

A20 tumor-bearing mice (n = 5/group) were treated with equal molar amounts of Erb-laIL2 (20 μg), anti-PD1 (10 μg) or PD1-laIL2 (20 μg) on day 17. Six days later, T cells from lymph nodes (LNs) and spleens were analyzed.

(**A**) $CD3^+$ T cell frequency in LNs from different groups is shown.

(**B**, **C**) $CD8^+$ T cell frequency in the LNs or spleen from different groups is shown separately in (**B**) and (**C**).

(**D**, **E**) $CD4^+$ T cell frequency in the LNs or spleen from different groups is shown separately in (**D**) and (**E**).

(**F**, **G**) The ratio of $CD8^+$ T cells to Treg cells in the LNs or spleen is shown separately in (**F**) and (**G**).

Data represent mean ± SEM. The *P* value was determined by one-way ANOVA with Tukey's multiple comparisons test (**A-G**).

Supplemental Figure 5. PD1-laIL2 causes the proliferation of PD1^{+}TIM3^{+}CD8^{+} effector T cells. (Related to Figure 5 and Figure 6)

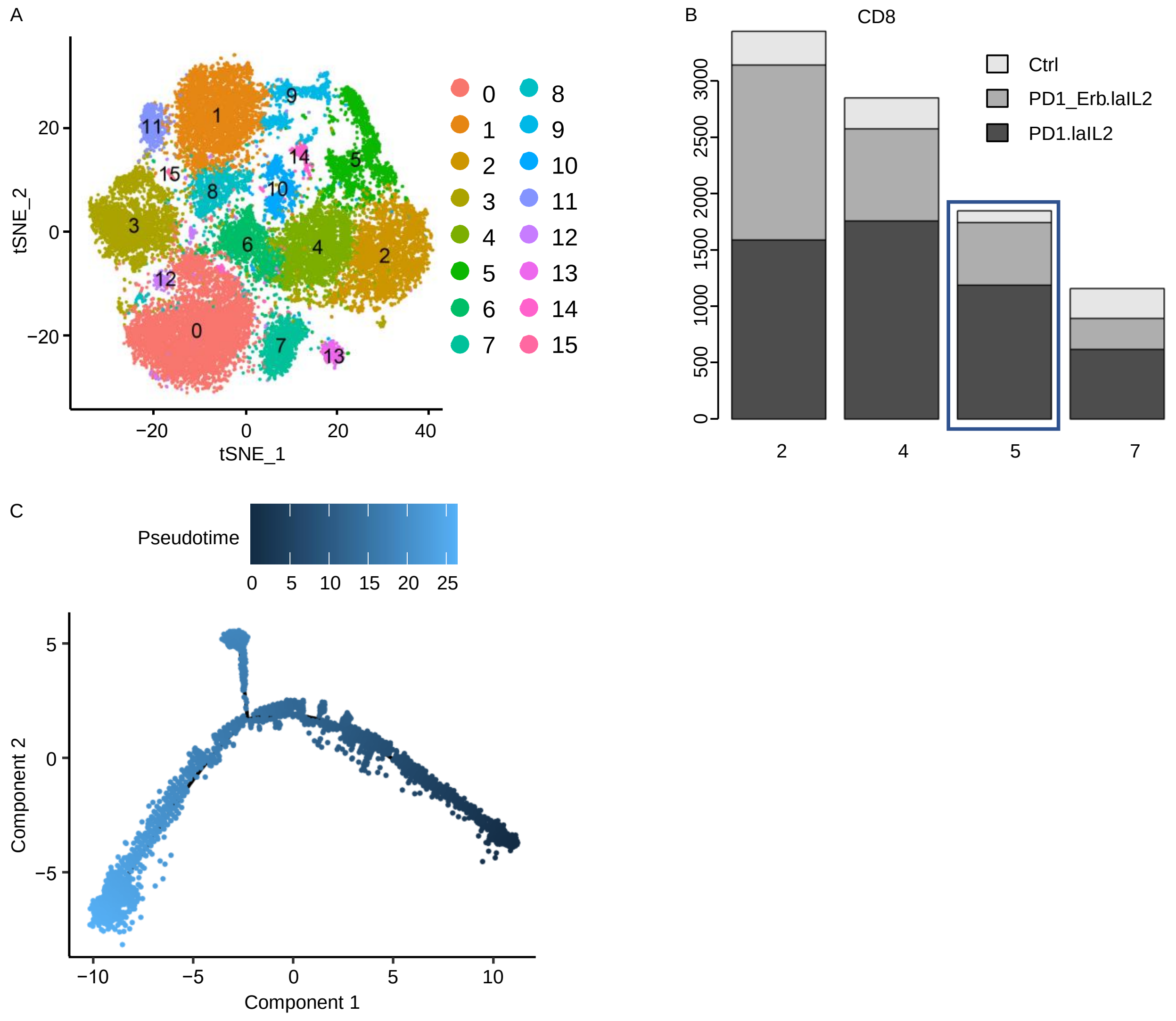

A20 tumor-bearing mice (n = 5/group) were treated with equal molar amounts of Erb-laIL2 (20 µg) and anti-PD1 (10 µg) (PD1_Erb.laIL2) or PD1-laIL2 (20 µg) on day 17. Three days later, CD3^{+} T cells from the tumor were sorted for single-cell RNA sequencing.

(**A**) T cells were separated into 16 clusters.

(**B**) Cell enrichment of each treatment group in CD8^{+} T cell clusters.

(**C**) Pseudotime analysis of CD8^{+} T cell clusters.

Supplemental Figure 6. PD1-laIL2 specifically reactivates PD1^{+}TIM3^{+} tumor-specific CD8^{+} T cells. (Related to Figure 7)

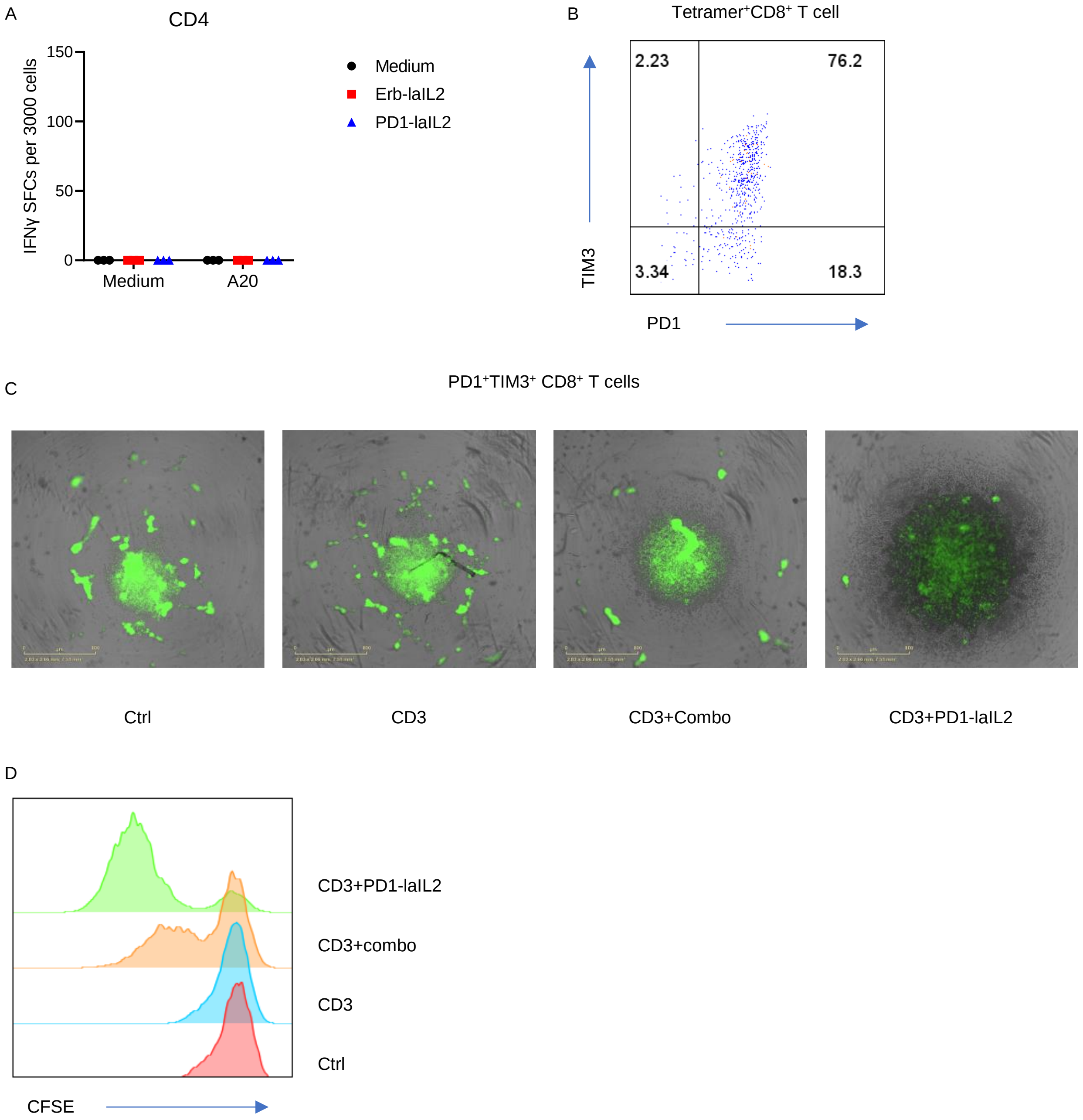

(**A**) CD4^{+}, PD1^{-}CD8^{+}, PD1^{+}TIM3^{-}CD8^{+} and PD1^{+}TIM3^{+}CD8^{+} T cells from A20 tumor-bearing mice were sorted out and cocultured with irradiated A20 cells in the presence of Erb-laIL2 or PD1-laIL2 for the IFNγ ELISPOT assay. Spots from CD4^{+} T cells are shown.

(**B**) Representative PD1 and TIM3 expressions on tetramer^{+}CD8^{+} T cells in tumors from MC38 tumor-bearing mice.

(**C, D**) Splenocytes were stimulated with anti-CD3 and anti-CD28 antibodies. Five days later, PD1^{+}TIM3^{+}CD8^{+} T cells were sorted out and labeled with CFSE. Then, the cells were cultured in 96-well plates in the presence of anti-CD3, Erb-laIL2 plus anti-PD1 (combo) or PD1-laIL2 for two days. The T cell clusters and CFSE brightness were assayed with an Incucyte® system (**C**). The representative CFSE brightness is shown in (**D**).
